# Tear lipocalin, lysozyme and lactoferrin concentrations in postmenopausal women

**Published:** 2015

**Authors:** I Careba, A Chiva, M Totir, E Ungureanu, Sinziana Gradinaru

**Affiliations:** * Ophthalmology Department, ”Carol Davila” University of Medicine and Pharmacy, Bucharest, Romania; ** Anatomy Department, “Carol Davila” University of Medicine and Pharmacy, Bucharest, Romania; *** Ophthalmology Department, Regina Maria - Private Health Care Network, Bucharest, Romania; **** University Emergency Hospital, Bucharest, Romania

**Keywords:** dry eye, postmenopausal women, tear lipocalin, lysozyme, lactoferrin

## Abstract

**Rationale:** Among the most frequently encountered pathologies examined by the ophthalmologist is dry eye syndrome (DE), which can be discovered particularly in the elderly. The initial diagnosis of DE is of high importance, but also challenging. This is because the biochemical changes in the tear film often develop before any detectable signs.

**Objective:** In this study, the possible relationship between ocular symptomatology, tear volume and tear break-up time (TBUT) and lipocalin, lactoferrin and lysozyme concentrations in the tear film were explored in a group of symptomatic dry-eyed postmenopausal (PM) women compared to age-matched controls.

**Patients and methods:** Sixty-six healthy PM females with ages of at least 50 years were grouped in two homogeneous lots (by age, post-menopause, co-morbidities) of 33 females each, one lot presenting mild or moderate dry eye syndrome (DE) and one asymptomatic non-dry eye (NDE), based on their feedback to the Ocular Surface Disease Index (OSDI) questionnaire and noninvasive TBUT and Schirmer test results. Tears were collected via capillary tubes and an eye wash method. Tear lysozyme, lactoferrin and lipocalin concentrations were determined via electrophoresis.

**Results:** OSDI responses revealed 3 mild DE, 30 moderate DE and 33 NDE. The OSDI total score and sub scores for the DE group were significantly greater than for the NDE group (p < 0.001). The mild and moderate DE group exhibited significantly shorter TBUTs compared to NDE (p < 0.001). No difference in tear lysozyme or lipocalin concentrations was found between DE and NDE groups, irrespective of the tear collection method, but a significant difference was found in lactoferrin concentration (p<0.001). No significant correlations were found between symptoms or signs of DE compared to either lipocalin, lysozyme or lactoferrin concentrations.

**Discussion:** In a PM population, lipocalin and lysozyme are invariable, irrespective of the presence and severity of DE symptoms. However, lactoferrin shows a significant decrease. This is a comprehensive study of lipocalin, lactoferrin and lysozyme in dry-eyed PM women and our results suggested that lactoferrin could be used as a biomarker of DE in postmenopausal women.

**Abbreviations:** PM = postmenopausal; DE = dry eye disease; NDE = non-dry eye; ELISA = Enzyme-linked immunosorbent assay

## Introduction

Dry eye syndrome (DE) is among the most frequently encountered pathologies examined by the ophthalmologists worldwide. The prevalence fluctuates between 7.4% and 33.7% [**1**]. Dry eye is discovered in women more than men, particularly in the elderly [**2**–**5**]. Many epidemiological studies highlight increasing age, menopausal and postmenopausal women, chronic androgen deficiency and oral contraceptive treatment as the most important factors that affect tear secretion, meibomian gland function and goblet cell density leading to DE [**1**]. The complexity of pathological mechanisms and the multitude of risk factors advocate for the development of new tear biomarkers, which became a requirement in order to obtain an accurate diagnosis of DE.

The early diagnosis is of high importance because the biochemical changes usually develop before the noticeable signs and symptoms. Without treatment, DE could lead to serious complications with an important outcome on visual acuity and quality of life [**1**,**6**,**7**]. The classic tests for diagnosing DE (Schirmer test, tear break-up time and fluorescein staining) are not too precise [**8**,**9**]. The discovery of new tear biomarkers could be effective, but complications in measuring them limit their role as routine tests.

DE is a multifactorial disease in which the disproportionate evaporation and deficiency of tear production are considered the leading mechanisms that could operate separately or concomitantly [**10**,**11**]. International Dry Eye Workshop (DEWS) revised the definition of DE by including tear film hyperosmolarity and inflammation of the ocular surface in the evolution of the disease [**12**,**13**]. 

## Materials and methods

We conducted this study in compliance with good clinical and medical practice, institutional review board regulations, informed consent practice and the principals of the Declaration of Helsinki. This current paper uses as patients the subjects in our earlier published study [**14**], in which we only underlined partial conclusions. We also used the same inclusion and exclusion criteria [**14**]. The inclusion criteria for the participants were represented by women with natural menopause and non-Sjögren’s DE, ≥ 45 years of age; they were recruited from a single center. Subjects were considered postmenopausal (PM) if they had no menses for at least 12 months. Subjects were diagnosed with DE based on the following: 1) documented diagnosis from medical charts made by a medical care provider ≥ 6 months prior to study visit and 2) a documented history for ≥ 3 months of ocular discomfort complaints consistent with DE. Exclusion criteria for all subjects included: males, < 45 years of age, childbearing potential or menses within the last 12 months, surgical removal of ovaries with or without fallopian tube, removal of uterus or endometrial ablation, a medical diagnosis of Diabetes and/ or autoimmune connective tissue disease, Stevens-Johnson syndrome, keratorefractive ocular laser procedures, use of topical ocular medications, corneal surgery, punctal cauterization or current punctal plugs. In addition, patients with a history of contact lens wearing within the past 6 months or intraocular laser procedures within 1 year from the study visit were excluded. These patients were classified as normal - non-dry eye (NDE) subjects. After providing an informed consent, sixty-six patients were enrolled in the study, divided into two groups of 33 patients each, one group having DE and one control group NDE [**14**].

**Clinical Assessment**

Subjects were asked to answer a dry eye symptom questionnaire (OSDI) containing 12 questions which assessed the visual function, ocular symptoms and results of stressful environmental conditions [**11**]. 

**Sample Collection and Processing**

Unstimulated tears were collected by using a capillary tube after best-corrected visual acuity and slit lamp examination were measured. A graded disposable 5 μl microcapillary tube (Wiretol-Micropipettes, Drummond Scientific Co., Broomall, PA, USA) was employed with which up to 5 μl of tears/ eye were collected from the inferior temporal tear meniscus of each patient, without corneal anesthesia, ensuring that the surfaces were not touched during this process [**14**]. Tear biomarkers, such as lysozyme, lactoferrin and lipocalin concentrations were quantified via electrophoresis afterwards.

Routine clinical examination followed to further describe DE presence and severity. This included the measurement of tear breakup time (TBUT), ocular surface fluorescein staining, based on the NEI/ Industry workshop method [**10**,**14**]. Schirmer test with and without anesthesia was also done [**13**].

These criteria were used for DE diagnosis: OSDI score > 20 with one or more of the following signs: TBUT ≤ 10 seconds, punctate corneal fluorescein staining or Schirmer without anesthesia (Schirmer I) score < 10 mm [**13**,**14**]. 

**Tear biomarkers for DE**

Biochemical analysis of tear film significantly improved the diagnosis of DE, creating a differential diagnostic between aqueous tear deficiency and evaporative DE [**8**]. Many tear biomarkers are being tested employing a wide diversity of procedures, like electrophoresis, ELISA, high performance chromatography in thin layer, immunonephelometry. Tear osmolarity, electrophoresis and measurement of major tear proteins (lipocalins, lactoferrin and lysozyme) remain the most useful analysis [**8**,**14**,**15**]. Automated system Hyrys–Hydrasys SEBIA France used in agarose gel electrophoresis considerably augments the test’s resolution and sensitivity, even though SDS polyacrylamide gel electrophoresis measures the major tear proteins [**14**,**15**]. The main advantage is the identification and relative quantification of many proteins in a single test. The tamperings common to other types of electrophoresis (the human intervention in staining and destaining of electrophoregrams, the proteins absorption on filter paper or the reduced availability of concentration technique) are entirely erased by using SEBIA technique [**8**,**14**,**16**]. Only 5 μl of reflex unconcentrated tears are necessary for testing, collected in glass capillaries by using a non-invasive procedure.

Tear lipocalin (15–33% of the proteins in tears), a 17.45 kDa member of a family named lipocalins, is the main lipid carrier in human tears and is crucial in the ocular surface protection. Together with lysozyme, tear lipocalin is one of the most concentrated proteins in human tears. Tear lipocalin binds phospholipids and fatty acids from the cornea, their desiccation being avoided in this way [**14**,**17**]. Lipids are solubilized by tear lipocalin in the divalent cation rich milieu of the tear film, in this way lipocalin stimulating clarity and stability. The tear film stability and delay of evaporation are influenced by the integration of tear lipocalin into the Meibomian lipids at the aqueous-lipid-air interface [**14**,**18**,**19**]. 

Lactoferrin (24–27%) is a multi function chain polypeptide with properties such as anti-inflammatory, bacteriostatic and antioxidant. Lysozyme (44–47%) is a glycolytic enzyme with antimicrobial function [**20**]. These two tear components are detected on SEBIA electrophoregrams between the most important peaks [**15**] and are secreted by the acini of the main gland. They act as an indicator of a lacrimal gland function. Moreover, lower levels represent an important sign for an inflammatory response, low antioxidant function and also microbial infections predisposition (mostly lysozyme) [**14**,**15**]. 

## Results

As discussed in our previous paper [**14**], the ANOVA analysis was applied for the statistical analysis. A descriptive analysis (means, medians, standard deviations and range for continuous data and frequency analysis for categorical data) was used for all the target variables. Continuous quantitative variables are being used as averages +/- standard deviations, whilst categorical variables are presented as variables. When comparing continuous variables, the ANOVA analysis was used. A p value < 0.05 was considered to be statistically significant. In order to validate the association between the levels of biomarkers and dry eye disease, bivariate correlation analysis (Spearman or Person correlation coefficient calculation) was used.

Thirty-three PM females with DE were recruited into our study, with a selection based on recent diagnosis of DE. This is considered the DE group. Thirty-three normal, NDE PM females not using artificial tears or lubricants, were enrolled as described in the Methods, forming the second lot of patients - the NDE group.

As emphasized in our earlier poster in which partial results from this study [**21**] were presented, based on the general guidelines for OSDI test, all DE patients were found to fall into the mild-to-moderate DE category (3 patients in the mild DE category and 30 in the moderate DE category). The other 33 patients were considered NDE. The OSDI total score and sub scores for the DE group were found to be significantly greater than for the NDE group (p < 0.001). 

**Fig. 1:**
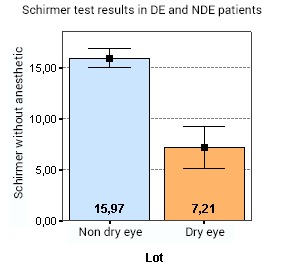
Shows that there is a statistically significant difference between the Schirmer test results in DE and NDE patients (p < 0.001). Thus, the levels of Schirmer tests are lower in the DE group, compared to the patients in the NDE group

The analysis of variance (ANOVA) showed that there is a statistically significant difference between the Schirmer test results in DE and NDE patients (p < 0.001) (**Fig. 1**). Thus, the levels of Schirmer tests are lower in the DE group, compared to the patients in the NDE group. 

**Fig. 2:**
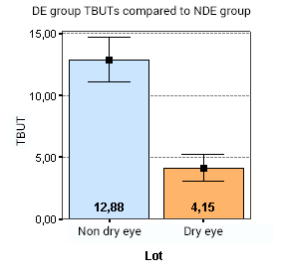
DE group exhibits significantly shorter TBUTs compared to NDE group (p < 0.001). While in the DE group mean TBUT is of 4,14 sec, mean TBUT in the NDE lot is of 12,88 sec

Also, the mild and moderate DE group exhibited a significantly shorter TBUTs compared to NDE group (p < 0.001) (**Fig. 2**). While in the DE group, the mean TBUT was of 4,14 sec, mean TBUT in the NDE lot was of 12,88 sec.

**Fig. 3:**
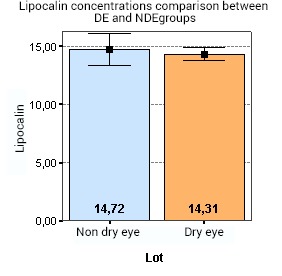
Shows no correlation between tear lipocalin (p=0,111) concentrations in the DE and NDE groups. Thus, tear lipocalin was in the same range in both groups

From the statistical data, no correlation was discovered between tear lysozyme or lipocalin (p=0,111) concentrations in the DE and NDE groups, irrespective of tear collection method. Thus, tear lipocalin and lysozyme were in the same range in both groups (**Fig. 3**).

**Fig. 4:**
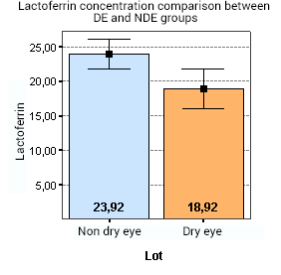
Shows that there is a statistically significant difference between lactoferrin concentration in the DE and NDE groups (p < 0.001). As it follows, in the DE group, a mean lactoferrin level of 18,92% was found, compared to 23,92% in the NDE group

The analysis of variance (ANOVA) showed that there is a statistically significant difference between the lactoferrin concentration in the DE and NDE groups (p < 0.001) (**Fig. 4**). As it follows, in the DE group, a mean lactoferrin level of 18,92% was found, compared to 23,92% in the NDE group.

No significant correlations were found between symptoms or signs of DE compared to either lipocalin, lysozyme or lactoferrin concentrations [**21**].

## Discussion

The levels of the three tear biomarkers analyzed (lactoferrin, lysozyme and lipocalin), were measured and some discrepancies between the patients with and without the presence of DE symptoms were observed.

In terms of differences between the 2 groups of patients, the following data was found, which was also presented in our previous poster [**21**]: the OSDI scores for the DE group were significantly greater than for the NDE group (p < 0.001). Also, the analysis showed that there was a statistically significant difference between the Schirmer test measurements in DE and NDE patients (p < 0.001). Thus, the levels of Schirmer tests are lower in the DE group, compared to the patients in the NDE group. Furthermore, the DE lot had significantly shorter TBUTs compared to the NDE group (p < 0.001). Whilst in the DE lot the mean TBUT was of 4,14 sec, the mean TBUT in the NDE lot was of 12,88 sec. Even though tear lipocalin and lysozyme were in the same range in both groups, showing no correlation between tear lysozyme or lipocalin (p=0,111) concentrations in the DE and NDE groups, the analysis of variance (ANOVA) demonstrated that there is statistically significant difference between lactoferrin concentration in the DE and NDE groups (p < 0.001). So, in the DE lot, the mean lactoferrin level was of 18,92%, compared to 23,92% in the NDE group. No significant correlations were found between symptoms or signs of DE compared to either lipocalin, lysozyme or lactoferrin concentrations [**21**].

These measurements highlighted the fact that there are alterations in the biochemical structure and constituents of the tears of DE patients, in comparison with the normal NDE patients, which could increase the chance of developing DE or intensify the underlying symptoms. The extent of these changes has been related to the severity of the syndrome. Our study can help in the better assessment of patients with DE and convey an extensive diagnosis and treatment for a concealed condition that may not be recognized by the patient, even though it may interfere with their quality of life and visual acuity.

To conclude, our results showed this was a study of lipocalin, lactoferrin and lysozyme in DE PM patients in which the electrophoresis of the tear film proteins was underlined by using automated system Hyrys-Hydrasys SEBIA France could become a significant investigation for the early diagnosis of the tear film changes, prevention and control of DE symptoms and consequences. 

**Acknowledgements**

This work received financial support through the project entitled “CERO – Career profile: Romanian Researcher”, grant number POSDRU/159/1.5/S/135760, cofinanced by the European Social Fund for Sectoral Operational Programme Human Resources Development 2007-2013.

**Sources of funding**

This work received financial support through the project entitled “CERO – Career profile: Romanian Researcher”, grant number POSDRU/159/1.5/S/135760, cofinanced by the European Social Fund for Sectoral Operational Programme Human Resources Development 2007-2013.

**Disclosures**

None
